# Towards extracellular vesicle delivery systems for tissue regeneration: material design at the molecular level

**DOI:** 10.20517/evcna.2022.37

**Published:** 2022-10-14

**Authors:** Ao Chen, Hengli Tian, Nana Yang, Zhijun Zhang, Guo-Yuan Yang, Wenguo Cui, Yaohui Tang

**Affiliations:** ^1^Shanghai Jiao Tong Affiliated Sixth People’s Hospital, School of Biomedical Engineering, Shanghai Jiao Tong University, Shanghai 200030, China.; ^2^School of Bioscience and Technology, Weifang Medical University, Weifang 261053, Shandong, China.; ^3^Department of Orthopaedics, Shanghai Key Laboratory for Prevention and Treatment of Bone and Joint Diseases, Shanghai Institute of Traumatology and Orthopaedics, Ruijin Hospital, Shanghai Jiao Tong University School of Medicine, Shanghai 200025, China.

**Keywords:** Biomaterials, material design, extracellular vesicle delivery, tissue regeneration

## Abstract

The discovery and development of extracellular vesicles in tissue engineering have shown great potential for tissue regenerative therapies. However, their vesicle nature requires dosage-dependent administration and efficient interactions with recipient cells. Researchers have resorted to biomaterials for localized and sustained delivery of extracellular vesicles to the targeted cells, but not much emphasis has been paid on the design of the materials, which deeply impacts their molecular interactions with the loaded extracellular vesicles and subsequent delivery. Therefore, we present in this review a comprehensive survey of extracellular vesicle delivery systems from the viewpoint of material design at the molecular level. We start with general requirements of the materials and delve into different properties of delivery systems as a result of different designs, from material selections to processing strategies. Based on these differences, we analyzed the performance of extracellular vesicle delivery and tissue regeneration in representative studies. In light of the current missing links within the relationship of material structures, physicochemical properties and delivery performances, we provide perspectives on the interactions of materials and extracellular vesicles and the possible extension of materials. This review aims to be a strategic enlightenment for the future design of extracellular vesicle delivery systems to facilitate their translation from basic science to clinical applications.

## INTRODUCTION

Tissue regeneration is key to wound healing. Different conditions of tissue regeneration can lead to extremes from full necrosis to near-full recovery, during which regenerative therapies are highly critical, especially for severe injuries. For example, biomolecules, such as growth factors, cytokines^[1]^ and microRNAs (miRNAs)^[2]^, have been extensively explored. These signaling molecules direct intrinsic cells to attach, proliferate and differentiate into objective phenotypes. However, the administration of free biomolecules suffers from the shortcomings of fast degradation and consequent deactivation. Despite the use of delivery systems to ease the degradation problem, the paucity of biomolecules can lead to incomplete progression towards near-full recovery, unless complicated recombinant biomolecules are used^[3]^. Meanwhile, stem cell therapy by direct administration of exogenous stem cells into the injured tissue has also gained much attention. The full functions of the stem cells are expected to differentiate and replace the damaged tissue. However, the transplantation of stem cells alone cannot meet regenerative purposes due to the poor viability and diminished regenerative activity of the cells^[4]^. Such therapy can also induce immunogenicity of the body and is associated with possible ethical problems^[5]^. The discovery of extracellular vesicles (EVs) in 1967^[6]^ and further investigations in 1981^[7]^ revealed a new understanding of the communication mechanisms between cells. The EVs secreted by stem cells can act as their functional replicates and aid in the repair and protection of damaged tissue. These advances in understanding have generated research interest in using stem cell-derived EVs as intercellular vectors for regenerative therapy^[8]^. The use of EVs, therefore, takes the frontier in the development of tissue engineering, covering the regeneration of injured osteopathic^[9]^, neural^[10]^, craniofacial^[11]^, and myocardial tissues^[12]^. Nevertheless, EVs are still essentially assemblies surrounded by cellular membranes and thus have to be endocytosed or contacted by the target cells to function. With this concern, four challenges exist in the use of EVs in regenerative therapies:

1) Successful delivery of EVs to the injured tissue and interactions with the target cells;

2) Long retention of EVs in the injured tissue and protection from fast clearance by organisms;

3) The dosage of the delivered EVs above a desired threshold;

4) Preservation of the bioactivity of the EVs before delivery.

The exploitation of advanced materials in tissue engineering for the delivery of drugs, biomolecules and stem cells enlightened the solution to the above challenges, which led to the rapid advancement and high effectiveness of EV delivery systems. This is reflected by many recent representative reviews of the investigations. Akbari *et al.* surveyed some successful cases of hydrogel-associated EV delivery and pointed out the critical role of the hydrogels in keeping the EVs and the cargoes within stable^[13]^. They also discerned the existing problems for the optimal approach of quantitative loading of EVs and the suitable selection of materials. Huang and coauthors focused on the procedures of the preparation of a diverse library of EV-laden scaffolds, and listed the challenges to optimally loading EVs and determining the release profile of EVs, especially *in vivo*^[14]^. Riau and colleagues provided detailed information on the approaches to loading EVs into materials and the duration of release from different delivery systems^[15]^. The dosage of delivery, application of artificial materials and smartness of the delivery systems were stated by the authors as perspectives. Holkar *et al.* provided an overview of EV-laden materials for the recovery of bone repair and presented a library of different delivery systems so far^[16]^. Tsintou and coauthors reviewed the cases of injectable hydrogels for prolonged delivery of EVs to achieve maximized regeneration of motor function after ischemic stroke^[17]^. The authors gave high remarks on EV delivery systems and urged for a translation of such material in the future. Recently, Murali and Holmes provided detailed lists of the latest EV delivery systems, their corresponding *in vitro *release behaviors and their regenerative performances on specific disease models^[18]^. The authors also pointed out issues to be addressed for the clinical translation of the delivery systems, including optimization of material designs for specific diseases, in-depth understanding of *in vivo* release performances, detecting possible sources affecting EVs, and resolving EV manufacturing and storage problems. In their final comments, the authors suggested a better understanding of mechanisms of action and exploration of possible optimization approaches for future EV delivery systems.

From the above excellent reviews, it can be seen that most reviews are based on the viewpoint of the therapeutic performance of EV delivery systems. In fact, the determinants of EV delivery systems are the physicochemical characteristics and structures of the materials, which govern the overall delivery performance. Therefore, we present in this review a comprehensive survey of EV delivery systems from the viewpoint of material design at the molecular level by focusing on the relationship between material structures, physicochemical properties and the performance of EV delivery. Here, “the material” is used to denote the state without EVs, and “the delivery system” stands for the state with EVs loaded. We proceeded from general to specific with the characteristics of different delivery systems as a result of material designs based on various selections of molecules and processing strategies. The materials are further classified into bulk materials and scaffolds based on the modes of loading and delivery of EVs. The according EV loading and delivery behaviors, including the release profiles *in vitro*, retention of EVs *in vivo* and the resulting tissue regeneration, were extracted from representative investigations and analyzed in correlation to the change of materials at the molecular level. From the discussions of these aspects, perspectives were also given in consideration to the current missing links in light of material structures, physicochemical properties and the performance of EV delivery, with possible extensions of materials for future designs of EV delivery systems.

## WHAT ARE EXTRACELLULAR VESICLES?

Extracellular vesicles are essentially vesicles with lipid bilayer membrane structures but without functional nuclei, secreted by almost all types of organisms and cell types^[19-25]^. Although no consensus was reached for the assignment of EVs to particular biogenesis pathways or subtypes^[19]^, physical properties such as physical sizes can be used to generally classify the EVs, with diameters < 200 nm as small EVs and > 200 nm all the way to 2000 nm as large ones^[16,19]^. EVs carry cargoes from their parental cells^[16]^, including proteins, RNAs, DNA and cytokines, to direct the behaviors of target cells^[26,27]^ with promoted stability and lower immunogenicity. The cargoes can also be loaded through various engineering technologies to achieve specific therapeutic purposes^[28]^. Thereby, stem cell-derived EVs are a better choice for regenerative therapy compared to the direct use of parental stem cells^[29]^.

The preparation of EVs from cells is basically composed of three steps. The first is to allow the cells to secrete EVs. The conditions of growth of the cells and the cellular status have strong impacts on EVs^[30-32]^. Hence the correct status of the cells must be secured for the production of desired EVs. This step is followed by the isolation of EVs from the culture media of the cells^[16]^. Common approaches are primarily based on physical separation by the distinction in specific physical properties of EVs, including differential ultracentrifugation, density gradient centrifugation, size exclusion chromatography, ultrafiltration, flow field-flow fractionation and microchip-based techniques^[16]^. Some chemical or immunological based methods have also been applied, such as polymer-based precipitation, chemical-based precipitation, immunoprecipitation and immunoaffinity capture^[16]^. Identifications of morphological properties (electron microscopy), size (dynamic light scattering, nanoparticle tracking analysis or tunable resistive pulse sensing), surface protein markers, and proteomic and RNA cargoes are usually conducted after isolation to confirm the successful harvest of EVs^[33,34]^. The isolated EVs are finally cryo-preserved within antifreeze agents or lyophilized with trehalose for storage^[33]^.

EVs have to interact with the recipient cells to exert functions, either by fusion and releasing the cargoes, initiation of downstream signaling cascade, or endocytosis after binding of EVs with the cells^[8]^. The cargoes of the EVs are then transferred to the recipient cells to construct crosstalk from the parental cells at molecular level, including immunomodulation, anti-inflammation, angiogenesis and specific direction of differentiation. This helps the levitation of harsh microenvironment and facilitates the recovery of the injured tissue. Nevertheless, the efficiency of such therapy is still low as simple systemic injections of EVs cannot guarantee the reception of EVs by the targeted cells despite the engineering of targeting function towards EVs. This is reflected by two factors. One is that the EVs fail to reach the recipient cells after systemic administration and are quickly cleared out from body^[35]^, while the other is the insufficient duration of the supply of EVs, leading to degradation of most EVs by lysosome in a single administration^[36]^. A localized and prolonged supply of EVs in the injured tissue can ensure the delivery to the recipient cells at optimal effect. With the introduction of materials for EV delivery, such vesicles can be retained for a longer time and at sustained concentration for localized delivery.

## ROLES AND REQUIREMENTS FOR EV DELIVERY SYSTEMS

In order to localize the delivery of EVs to the cells in the injured tissue and extend the whole process, the EV delivery systems are exploited to carry the EVs, transplanted and filled within the injury. Being an agent between the tissue and the EVs, the delivery systems form two interfaces with the EVs and the tissue, taking two roles oriented to each of the interfaces.

Towards the tissue, the material fills the gap created by the injury and provides mechanistic support for cells to attach after their reception of the EVs; it later degrades (if designed to do so) to provide space for further growth of new tissue. The requirements of the EV delivery systems for tissue are as follows:

1) Localized delivery of EVs: the material is applied within the gap of injury to ensure delivery of EVs to recipient cells within the local tissue;

2) Biocompatibility: the material, including the possible product of degradation, should be non-irritating, non-toxic, non-inflammatory and non-carcinogenic to the local tissue;

3) Mechanical support: the material serves as the initial attachment point of cells to migrate, proliferate and differentiate into new tissue;

4) Degradability: the material designed to be degradable should degrade either through hydrolysis, responsive decomposition or biodegradation to provide sufficient space for the regeneration of tissue;

5) Other detailed needs: based on the site of application of the material, including but not limited to mechanical strength (for bones), elasticity (blood vessel and muscle), optical properties (cornea), *etc*.

**Figure 1:**
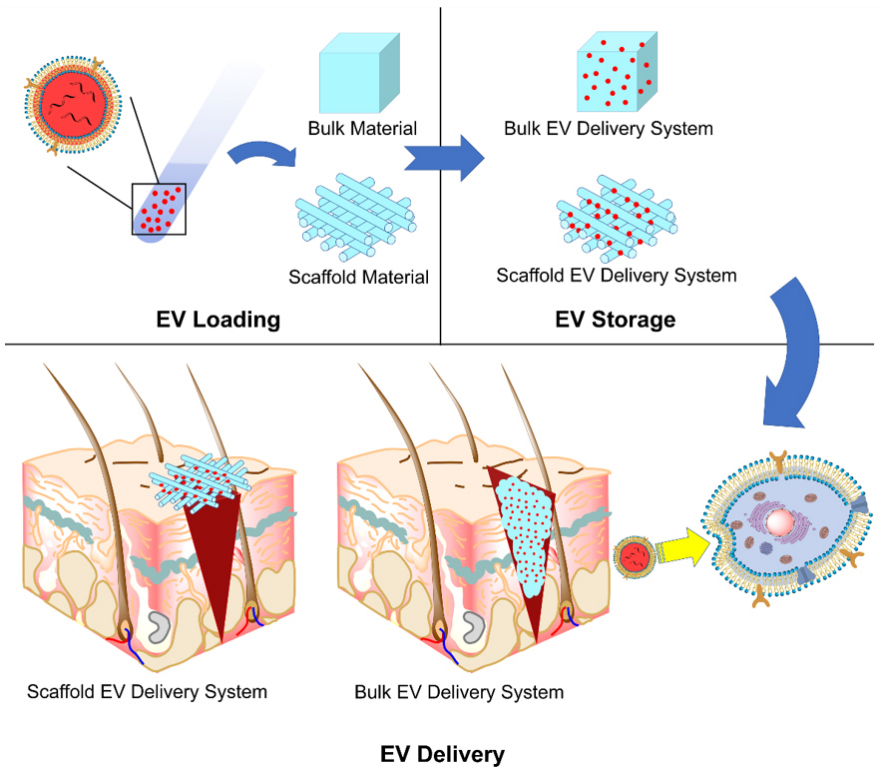
Schematic illustration of the roles of EV delivery systems: EV loading, EV storage and EV delivery. EV: Extracellular vesicle.

The role towards the EVs is the relay of EVs towards the recipient cells, which is initiated by loading, continued by storage and ended by the delivery of EVs [Figure 1]. The requirements of each step should be met to assure that the EVs reach the target:

1) EV loading: EVs should be retained within or immobilized upon the material at a high loading efficiency, with no or low possibility of release before transplantation;

2) EV storage: Upon being loaded, EVs should be well protected from possible damages, including physical (primarily mechanical) and chemical (primarily from the residue of chemicals used in fabrication) ones that will cause disruption of the vesicles;

3) EV delivery: The delivery should be to the recipient cells at a constant rate and match the kinetics of tissue regeneration. Too fast or too slow of delivery will result in either overflow or insufficient dose, both leading to deficiency of the therapy.

## DESIGN STRATEGIES OF MATERIALS FOR EV DELIVERY SYSTEMS

### General physicochemical properties of EV delivery systems

As the interface towards the tissue to regenerate, EV delivery systems vary with different choices of materials and functions. The key physicochemical characteristics of materials as the interface for cells are mainly focused on the mechanical and surface properties^[37]^. The mechanical strengths of the material can be classified into compression, tension, torsion and shearing moduli according to the directions of force, which are critical mechanical cues towards the proliferation and differentiation of stem cells and further the regeneration of new tissue^[38]^. For example, a hydrogel for brain tissue is required to be approximately 180 Pa in shearing modulus, similar to that of brain tissue, for neural stem cells to successfully proliferate and differentiate into neurons^[38]^, while bone tissue requires hydrogels with compression moduli of 225 kPa to support osteogenesis^[39]^. When the attachment of cells occurs, the surface properties of the materials come first for the cells to sense, including the surface tension and the surface charge. Different opinions exist regarding the optimal surface tension for the attachment of cells, but it is considered that both superhydrophilic (water contact angles < 30 °) and superhydrophobic (water contact angles > 150 °) surfaces are improper for cell adhesion^[40]^. For surface charge, electrostatic interactions of positively charged surfaces can enhance the sedimentation of negatively charged cells, while the negatively charged surfaces can improve the adsorption of proteins for more binding of the cells^[41]^. The use of specific proteins, such as integrin, to modify the surfaces can also promote specific binding of the cells to the surfaces^[40]^. All of the above physicochemical properties take different roles in regulating the active changes of the cells in the injured tissue and can be achieved by carefully selecting materials or modifying their structures.

On the other side of the interface, the incorporated EVs have similar membrane structures to their parental stem cells, so the basic surface properties of the material still apply to them. Nevertheless, EVs do not possess cellular entities to actively start an attachment procedure as cells. Therefore, specific attention to the design of the material, especially for molecular sources and processing strategies, must be paid to secure the interactions between materials and EVs for both loading and delivery. As long as the designated biomedical properties are fulfilled, the selection of materials is non-specific to the EVs used.

### EV delivery systems from polymeric sources

#### Water-soluble polymer-based bulk hydrogels for EV delivery systems

Hydrogels are bulk materials formed by crosslinked water-soluble polymers. Thus crosslinking is the molecular symbol of such materials. The meshes formed by the crosslinked polymer chains act as niches for the encapsulation and retention of biomolecules, as reflected by the vast studies of hydrogels as drug delivery systems^[42]^ for different parts of body, and this is now extended to the delivery of EVs. The sizes of the meshes are mainly controlled by the density of crosslinking, including not only the number of chemical or physical crosslinking junctions in a unit volume of hydrogel but also the physical entanglement of the polymer chains, which increases with the concentration and the lengths (molecular weights) of the polymer chains. A higher crosslinking density limits the swelling ratio of the hydrogel by reducing the entropy after the swelling process^[43]^, limiting the sizes of the meshes, increasing the number of meshes and raising the overall mechanical strengths of the hydrogel. By adjusting the crosslinking density from low to high, the mesh size can cover the order of 1 nm to 100 nm^[43]^, and the elastic moduli range from Pa to MPa levels accordingly. Such easy customizability in both structure and mechanical properties makes hydrogels versatile to be competitive for different types of tissues, especially soft tissues such as neural and cerebral tissues^[44]^. The surface property of hydrogels is fundamentally hydrophilic, creating a water-abundant surface resembling that of the tissue surface and therefore improving their compatibility with EVs and coming cells. In addition, most water-soluble polymers for hydrogels can form strong hydrogen bonds with the membrane proteins of cells. In some cases, positively charged hydrogels can promote the attachment of the recipient cells of the EVs. A good example is chitosan hydrogels with protonated ammonium moieties^[45-47]^.

Most hydrogels exploit natural water-soluble polymers as major components due to their inherent biodegradability. Reported natural water-soluble polymers include pullulan^[48]^, chitosan^[47,49]^, silk fibroin^[50]^, cellulose^[51]^, alginate^[50]^, gelatin^[52]^ and hyaluronic acid^[53,54]^. Among them, gelatin and hyaluronic acid are water-soluble extracts of decellularized extracellular matrices (ECM) and are highly compatible with EVs due to their close nature to ECM. These natural polymers are biodegradable, which is crucial for bulk hydrogels to deliver EVs, and some of them have special intrinsic properties, such as bacteriostatic and hemostatic chitosan. For water-soluble artificial polymers, past studies have mainly focused on relatively limited choices of polymers, such as polyol series [poly(ethylene glycol)(PEG)^[55]^ and Pluronic F-127^[56,57]^], polyethyleneimine (PEI)^[48]^, vinyl polymer [mostly poly(vinyl alcohol) (PVA)]^[57,58]^ and artificial peptides^[59]^. These polymers have specific functions based on their special structures, but are non-biodegradable except for artificial peptides. Therefore, in many cases, both artificial and natural polymers have been used to provide specific functions and overcome the shortcomings of hydrogels from a single source.

Crosslinking of the above materials is achieved through modification of the polymers or the use of special crosslinkers. Through different crosslinking structures, the hydrogel EV delivery systems can be classified and special properties be endowed. Table 1 lists the crosslinking strategies of hydrogels for EV delivery so far, including the molecular structures of the crosslinking junctions, representative materials and the corresponding properties. In addition to the single crosslinking structures shown in Table 1, some hydrogels incorporate double crosslinking network systems, especially trigger responsive ones, to achieve more controllable crosslinking and other intrinsic functions. The hydrogel developed by Li and colleagues exhibited both thermal and pH responsiveness derived from respectively Pluronic F-127 for and carboxymethyl chitosan-genipin to encapsulate human umbilical cord-mesenchymal stem cells (hUCMSCs)-derived exosomes for skin repair^[73]^. The thermal responsiveness leads to *in situ* crosslinking, while the aldehyde-amine crosslinking allows pH-responsive release of EVs to match the weakly acidic microenvironment of injuries. As for Guan and coauthors’ hydrogel, GelMA works as the main matrix and aldehyde-functionalized chondroitin mimics ECM in growth plate cartilage to supply ECM environment, promote cartilage healing with the help of bone marrow mesenchymal stem cell-derived EVs, and provide anti-inflammatory functions^[74]^. 

**Table 1:** Crosslinking strategies, suitable selections of materials and the corresponding properties of hydrogels used as EV delivery systems

**Crosslinking strategies and structures**		**Representative materials**	**Properties**
**(Chemical) Polymerization of methacrylamide or methacrylate**	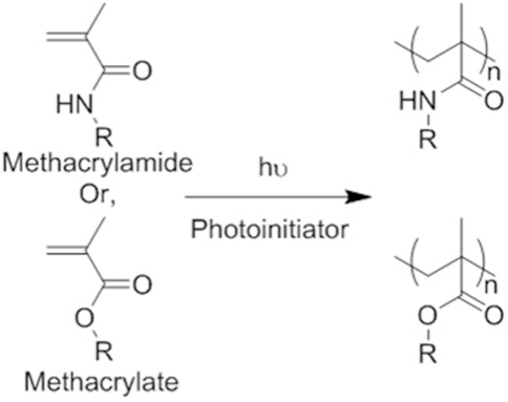	Gelatin methacrylamide (GelMA)^[52]^	*In situ* photo-crosslinking
		Hyaluronic acid methacrylate (HAMA)^[53,60]^	
**(Chemical) Thiol-ene reaction**	Free radical 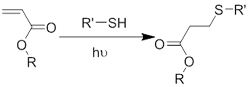	Poly(ethylene glycol) diacrylate/thiolated alginate^[61]^	*In situ* photo-crosslinking
	Retro Michael addition 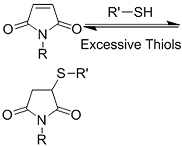	Miktoarm PEG respectively modified with telechelic thiol/maleic acid^[62]^	Injectable using double-barrel syringe *In situ* crosslinking
**(Chemical) Amine-aldehyde conjugation (formation of Schiff base)**	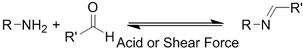 Introductions of aldehyde: *Oxidation of polysaccharides:* 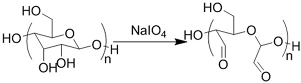 *UV radiation over o-nitrobenzyl groups (fast crosslinking):* 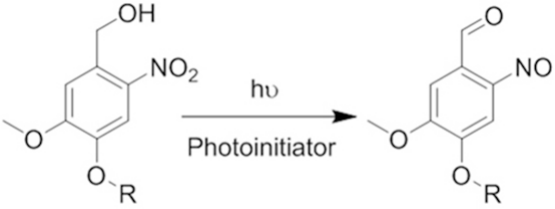 *Modification with 4-formylbenzoic acid:* 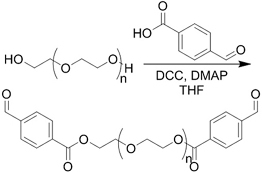 Introductions of amine: *Conjugate with hydrazine:* 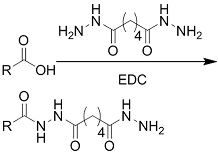	Oxidated pullulan and PEI-modified pluronic F-127^[48]^ Oxidated hyaluronic acid and poly-*ε*-L-lysine^[63]^ Oxidated methylcellulose and PEI modified Pluronic F-127^[51]^ *o*-nitrobenzyl alcohol modified hyaluronic acid and gelatin (UV responsive)^[64]^ Chitosan and diformalbenzoic acid-modified PEG^[65]^ Hyaluronic acid modified with adipic dihydrazide and oxidated alginate^[66,67]^	Injectable through shear-thinning Self-healing Tissue adhesive Acid responsive degradation (pH < 6)
**(Chemical) Phenylboronic acid-diol conjugation**	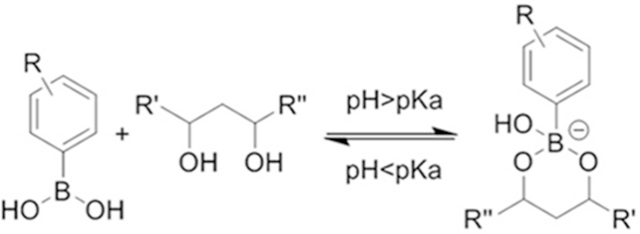 H_2_O_2_ responsiveness: 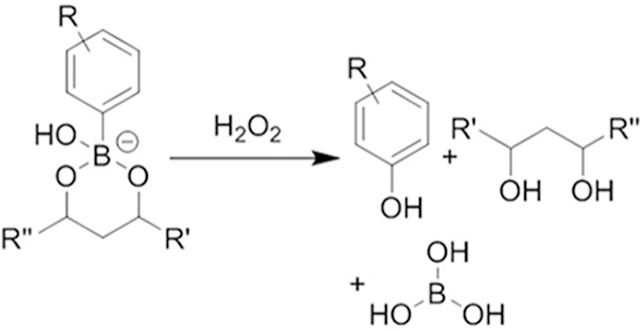 Glucose responsiveness: 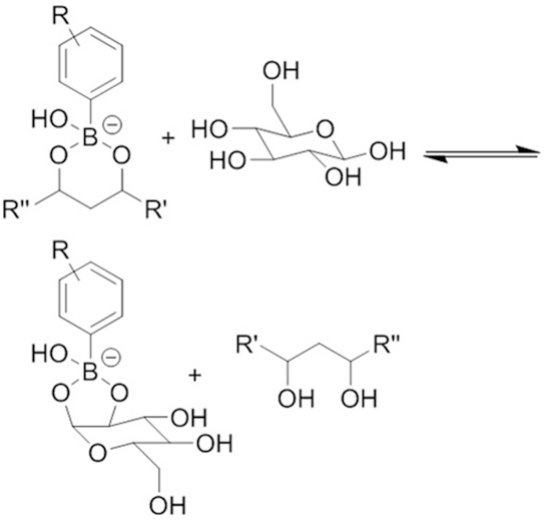	Hyaluronic acid modified with phenylboronic acid and PVA^#[68]^	Injectable through shear-thinning Self-healing Acid responsive degradation H_2_O_2 _responsive degradation Glucose responsive degradation
**(Chemical) Silver ion-thiol bonds**	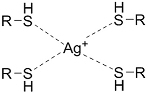	Silver ion and thiolated miktoarm PEG^[62]^	Injectable through shear-thinning Self-healing *In situ* crosslinking
**(Chemical) Enzyme catalysis of dopamine to form free radicals and chemical conjugation**	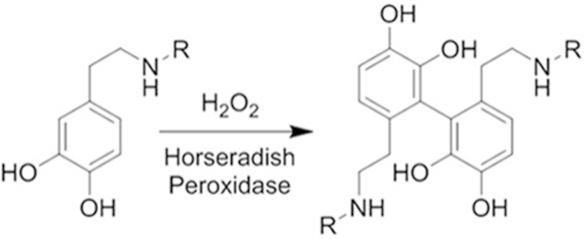	Dopamine-modified alginate catalyzed by H_2_O_2_ and horseradish peroxidase^[50]^	Injectable Tissue adhesive *In situ* crosslinking
**(Chemical) Enzyme catalysis of water-soluble protein to form insoluble clusters**	Structure varies	Partial catalysis of fibrinogen^#^ to fibrin by thrombin^#[69]^	Fast crosslinking, specific reaction
**(Physical) Electrostatic interactions**	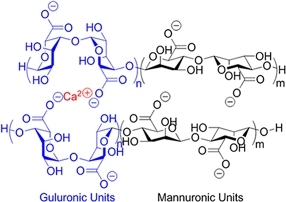	Alginate in aqueous solution of calcium salt^#[70]^	Submersion in Ca^2+^ solution for crosslinking
**(Physical) Temperature raising above LCST, forming hydrophobic clusters**	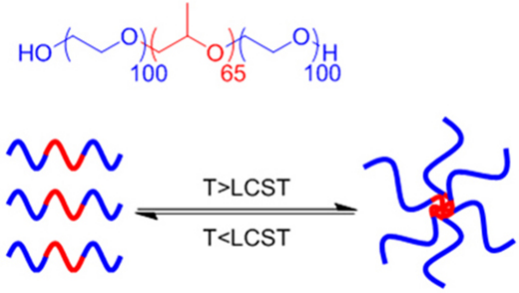	Pluronic F-127^#^ above 37 °C^[56]^	Injectable Temperature sensitive *in situ *crosslinking
**(Physical) Combination of electrostatic, hydrophobic and hydrogen bonding interactions**	Ionic shielding of electrostatic repulsion, enhancement of hydrophobic interaction, and redistribution of hydrogen bonding with increasing temperature	Chitosan^#^ and *β*-glycerophosphate^#^, gelation above 37 °C^[47]^	Injectable Temperature sensitive *in situ* crosslinking
**(Physical) Hydrogen bonds**	Peptides 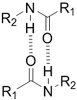	Arginine-Glycine-Aspartate (RGD)-Biotin^[59]^	Crosslinking after annealing
	PVA-PEG interaction 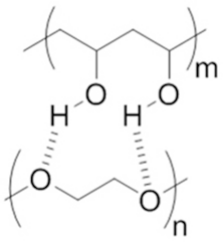	PVA^#^ and PEG^#^ blend hydrogel^[57]^	Extrusion of PVA into PEG solution
	Junction formation by end groups 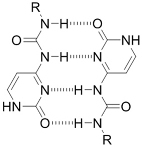	PEG with telechelic ureidopyrimidinone^[55]^	Injectable pH-responsive *in situ* crosslinking (basic to neutral)
**(Physical) Host-guest interactions**	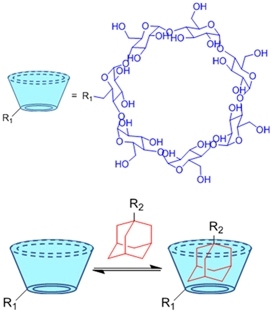	Adamantane- and *β*-cyclodextrin-modified hyaluronic acid^[71,72]^	Injectable through shear-thinning

^#^Commercially available biomaterials. EV: Extracellular vesicle; PEI: polyethyleneimine; PEG: poly(ethylene glycol); PVA: poly(vinyl alcohol); LCST: lower critical solution temperature.

With such a wide selection of polymers and crosslinking strategies, one can easily acquire hydrogels with desired properties. Such versatility makes hydrogel materials suitable for different injuries and desired regenerative therapies.

#### Insoluble polymer-based scaffolds for EV delivery systems

Polymeric scaffolds, in both 2D (membranes) and 3D (scaffolds) forms, serve as microporous matrices for cells to attach, proliferate, differentiate, and finally lead to the regeneration of tissue at the implanting area. The incorporation of EVs into the scaffolds can enhance the regenerative function. However, unlike hydrogels, the polymers for scaffolds are water-insoluble. Therefore, unless emulsion techniques are used (see “ADDITIONAL FUNCTIONS OF EV DELIVERY SYSTEMS”)^[75]^, it is improper for normal-sized scaffolds to encapsulate EVs within, but provide surfaces for EVs to attach and thereafter deliver them to the recipient cells. The water insolubility and high temperature of glass transition (*T_g_*) of the scaffold polymers lead to high elastic moduli easily reaching MPa level for both artificial polymeric scaffolds^[76]^ and natural polymer-derived scaffolds^[77]^, making them the choice of tissue engineering in parts requiring high tensile/compression strengths, such as bones^[78]^, bladders^[79]^ and dermis^[80]^.

The major difference between hydrogels and scaffolds is the delicate porous structure. Porosity defines the overall available space within the scaffolds. Higher porosity provides more surface area for the loading of EVs. The average pore size is another indicator of the accessibility of the scaffolds. Depending on the source of the material, different porosity-introducing procedures are used. The representative materials and the corresponding processing methods are listed in Table 2. Both the porosity and the pore size can be adjusted by the bottom-up fabrication of the material (through 3D printing) or the sizes of the media to induce porosity (through sacrificial templates). The surface wettability of scaffolds is more crucial than that of hydrogels since their interactions with EVs are mainly at the surface of the material. Due to the explicit hydrophobic functional groups, polymers for the fabrication of scaffolds are mildly hydrophobic on the surface (often with static water contact angles of 60-90 °), which is slightly above the optimal range of surface tension for the adsorption of EVs (with static water contact angles approximately 40-70 ° by referring to the attachment of cells)^[103]^. Thus, different surface wettability is tuned depending on the type of EV to reduce excessive hydrophobicity. Generally, the adjustment of surface wettability is through coating hydrophilic materials, especially positively charged polymers, to change the explicit functional groups and thereby promote the adsorption of cells and EVs (for details, see “IMPACT OF DIFFERENT DESIGNS ON THE LOADING AND DELIVERY OF EVs”).

**Table 2:** Representative water-insoluble polymeric materials used for EV delivery and typical approaches of porosity introduction

**Chemical nature**	Materials and structures (static water contact angle*)	**Approaches of porosity introduction**
**(Natural) Decellularized ECM**	Bovine bone^[81]^ (N/A)	Decellularization, followed by decalcification (bones), grinding, crosslinking and lyophilization
	Porcine cartilage^[82]^ (N/A)	
	Dermis of different animals^[83]^ (N/A)	
**(Natural) Protein**	Collagen^#[84,85]^ (~92 °)^[86]^	Electrospinning (in 1,1,1,3,3,3-hexafluoro-2-propanol (HFIP) or acetic acids) Sacrificial templates (cryogel)
	Silk Fibroin^#[87]^ (~74 °)^[88]^	
**(Natural) Polysaccharide**	Bacterial cellulose (membrane form only)^[77]^ (~33 °)^[89]^	Biofabrication by *Komagataeibacter xylinus*
**(Natural) Mixture of polysaccharide and protein**	Chitosan/silk fibroin^[90,91]^ (N/A)	Sacrificial templates (cryogel)
**(Artificial) Polyester**	Polycaprolactone (PCL)^#[92,93]^ (~75 °)^[94]^ 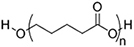 Poly(L-lactide) (PLLA)^#[95]^ (~80 °)^[96]^ 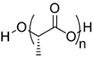 Poly(lactic-*co*-glycolic acid) (PLGA)^#[97]^ (57-78 °, from 5:5 to 9:1 LA: GA)^[98]^ 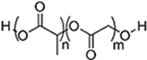	3D printing Sacrificial templates (sugar dissolution or cryogel) Electrospinning (in chloroform and dichloromethane, with polarity adjusted by *N*,*N*-dimethylformamide or methanol)
**(Artificial) Polyurethane**	Diol: ascorbic acid, PCL diol; Diisocyanide: hexamethylene diisocyanide^[99,100]^ (N/A) 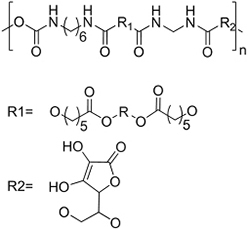	Sacrificial templates (cryogel)
**(Artificial) Others**	Polyetheretherketone^#[101]^ (~85 °)^[102]^ 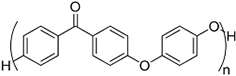	Strong acid corrosion

*Contact angles of materials without artificial introduction of surface topography. ^#^Commercially available biomaterials. EV: Extracellular vesicle; ECM: extracellular matrices.

The versatility of scaffolds, unlike the wide selection of materials and crosslinking mechanisms for hydrogel, lies in the different porous structures. Changes in pore sizes, pore shapes, and connectivity of the pores, may lead to various EV loading situations and subsequently, different cell attachment, migration and communication. With fine control over the porosity-introducing, different porous structures can be further investigated for optimized regenerative results.

#### Hydrogel scaffolds for EV delivery systems

Hydrogels are materials encapsulating EVs, serving as bulk materials to fill the space of injuries. They have adjustable crosslinking density to precisely control the mechanical properties in resemblance to tissues, but have low surface areas for the attachment of cells for regeneration. On the other hand, scaffolds have delicate porous structures providing a large surface area for the attachment of EVs and the attachment, migration, proliferation and differentiation of cells, but the high mechanical strength limits their area of application. The combination of hydrogel and scaffold structure into hydrogel scaffolds can merge the advantages while overcoming the shortcomings. 

Based on the definition of scaffolds, shear-thinning hydrogels, which self-heal after a certain period of time, are not suitable for the fabrication of hydrogel scaffolds. *In situ* photocrosslinkable hydrogels such as GelMA^[104]^ and alginate methacrylate (AlgMA)^[105]^ are the most popular choices. Similar to the fabrication of scaffolds, porosity can also be introduced through sacrificial templates or fine structure building (e.g., 3D printing). Due to the aqueous state of the polymer, ice crystals are the main choice for the templates. The overall procedure follows the route of solution freezing, photocuring and lyophilization^[91]^. With the swelling property, lyophilized hydrogel scaffolds exhibit a water-absorbing function when submerged in EV suspensions, providing the potential to suck EVs into the pores distributed on the surface. At the same time, pores become smaller following swelling so that the absorbed EVs are enclosed in the pores. Such behavior gives them the name of “hydrogel sponges” for the loading of EVs through absorption^[91]^. Shi *et al.* developed a chitosan (low degree of deacetylation)/silk hydrogel sponge to load EVs from gingival mesenchymal stem cells, with silk fibroin providing mechanical support, and chitosan performing the swelling function and providing cationic charges^[91]^. This hydrogel scaffold overcame the excessive swelling of sole chitosan and the brittleness of sole fibroin scaffolds. Similarly, a triple-layer hydrogel scaffold composed of bladder acellular matrix, partially oxidated alginate-grafted gelatin hydrogel (crosslinked by aldehyde-amine and calcium ions) and knitted silk mesh was designed by Xiao and colleagues to provide sufficient waterproofness and stretching resistance^[106]^. Such a structure is similar to “steel and concrete”, providing strong mechanical properties in resemblance to those of bladder.

The bottom-up 3D printing of polymer solution with simultaneous printing and crosslinking can build well-defined hydrogel scaffolds. Due to the overall non-damaging process, the EVs can be loaded either after the fabrication of scaffolds as in ordinary fabrication procedure, or can be directly mixed in the polymer solution during the preparation of bulk hydrogels, which is called “bioink”. Chen and colleagues developed a 3D-printable bioink composed of GelMA, mesenchymal stem cell-derived EVs and porcine cartilage ECM powder^[104]^. The resulting hydrogel scaffolds, therefore, acquired radially oriented 3D architectures as designed, which facilitated the cell ingrowth for the regeneration of cartilage with the help of mesenchymal stem cell (MSC) derived EVs. Materials of other sources, such as alginate-Ca^2+^ hydrogels, which do not possess *in situ* photo-crosslinking functions but require the addition of specific crosslinking molecules, can undergo monolayer printing-Ca^2+^ solution crosslinking cycles to achieve a multi-layered scaffold, as demonstrated by Sun *et al.* in their macrophage EV-laden alginate scaffolds for osteogenesis and angiogenesis^[107]^. 

The combination of hydrogels and scaffolds brings great diversity to the design and fabrication of EV delivery systems. This endows future EV delivery systems with high tailorability for any injury at any location.

### EV delivery systems from inorganic sources

Unlike polymeric materials, inorganic materials are essential minerals with high elastic moduli. For example, hydroxyapatite, with a formula of Ca_10_(PO_4_)_6_(OH)_2_, is the major inorganic component of bones, whose elastic modulus can reach ~150 GPa in the form of single crystals under nanoindentation^[108]^. In consideration of the type of tissue with high stiffness and with a high abundance of mineral contents, inorganic material EV delivery systems can and have only been applied for bone regeneration. The commonly utilized inorganic materials include *β*-tricalcium phosphate [*β*-Ca_3_(PO_4_)_2_, *β*-TCP] ceramics^[109]^, titanium oxide^[110]^, titanium alloy^[111]^, bioactive glass (45S5)^[112]^, hydroxyapatite^[113]^, and calcium sulfate^[113]^. These materials have been used as bone substitutes to exert osteoinductive and osteoconductive functions in tissue engineering for years, especially hydroxyapatite, the major inorganic component of bones. Some of the above materials have to be specifically processed from the raw materials to achieve the desired compounds or structure. 

Inorganic materials can be used as bulk materials for the encapsulation of EVs and scaffolds for the attachment of EVs. With their low solubility, a slurry of salts and EVs can set into solid bulk forms after slight evaporation of excessive water. Qayoom and colleagues reported such a bulk salt EV delivery system fabricated by the setting of nanohydroxyapatite, calcium sulfate, human bone morphogenetic protein-2, zoledronate and bone marrow mesenchymal cell-derived EVs^[113]^. The slow dissolution of calcium sulfate (~0.26 g/100 g water at 25 °C for CaSO_4_·2H_2_O, decrease with temperature^[114]^) allows slow creation of pores and release of encapsulated EVs while providing space for the attachment and infiltration of cells. However, compared to the mode of bulk materials, more popularity lies in the mode of scaffolds in the exploitation of inorganic materials. The porosity of the scaffolds can also be introduced, again, using sacrificial templates and 3D printing. Unlike polymeric scaffolds with ice or sugar as sacrificial templates, inorganic scaffolds use polymer beads since a high-temperature sintering process is required to merge the minerals together and mold the shape. The bioglass scaffolds developed by Liu and colleagues used different polymeric templates to introduce hierarchical pores: micelle-forming Pluronic F-127 to form mesopores (7.7 nm), microspheres (0.5-2 μm) to form micropores and polyurethane sponges to form macropores (200-500 μm)^[115]^. All materials were mixed with mineral slurry to set and sintered to merge and burn off the polymeric templates to achieve hierarchically porous structures for the attachment of mesenchymal stem cell-derived exosomes. A similar process has been applied to commercially available porous *β*-TCP scaffolds^[109,116]^. 3D printing can combine the steps of sintering and structure-building, as demonstrated by the Ti_6_Al_4_V alloy and lithium chloride incorporated bioglass scaffolds prepared by Zhai *et al.*^[111]^ and Liu *et al.*^[112]^ for the attachment of EVs through incubation. For pure metals with even higher melting points, which cannot be processed by sintering or 3D printing, an even stronger method is needed. Titanium must be processed by electrochemical anodization to fabricate nanotube topography on the surface as a 2D scaffold for further attachment of EVs and bone regeneration^[110]^. Again, most inorganic scaffolds are treated with coatings to change the surface chemistry at the molecular level and enhance the interaction with EVs.

Compared to polymeric materials, not many inorganic materials have been tested. The primary difficulties are the relatively harsh processing procedures and the narrow window of selection. It is expected that more inorganic materials with unique properties, such as 2D materials, can be explored in the future.

### EV delivery systems complexed of polymeric and inorganic materials

Most inorganic scaffolds have to be fabricated through sintering to achieve a certain volume for implantation, which requires well-controlled high temperatures. Otherwise, the raw materials are in powder form. In addition, the high stiffness of the material may not have a strong affinity for cells to attach and proliferate. Under such circumstances, the combination of polymeric matrix and inorganic material powder into complex material is favored to incorporate the advantages of both types of materials, in a way to take advantage of the maneuverability, degradability and affinity of polymeric hydrogels or scaffolds and the easy absorption of powder formed minerals by the bones. Such an approach is called “mineral-doping”. Chen *et al.* doped hydroxyapatite in thiolated hyaluronic acid and thiolated heparin hydrogel crosslinked by “ene” modified PEG^[117]^. This hydrogel was embedded with miRNA-375-carrying EVs from bone marrow mesenchymal stem cells to treat calvarial defects. Hydroxyapatite was also encapsulated within the hydrogel so that the injured bones could take advantage of the osteoinductive function for regeneration. For non-water-soluble scaffolds, Gandolfi and colleagues mixed finely milled dicalcium phosphate dihydrate and calcium silicate into PLA solution^[95]^. Through a phase separation technique, which involves the freezing of a polymer solution and the use of a miscible solvent with an even lower melting point to remove the frozen solvent^[118]^, a mineral-doped PLA porous scaffold was obtained, with pore diameters ranging from 10 to 200 μm. The minerals, in the form of fine powder, are easier for osteoinduction. This scaffold was later surface-attached with EVs from mesenchymal stem cells to achieve full osteogenic function for bone regeneration. With the development of both polymeric and inorganic materials, more combinations and more functions can be expected for complex EV delivery systems.

## IMPACT OF DIFFERENT DESIGNS ON THE LOADING AND DELIVERY OF EVs

### For bulk materials: EV-encapsulation and release

For bulk hydrogels (including hydrogel scaffolds fabricated by the 3D printing of EV-encapsulated bioink^[104]^) or bulk inorganic materials, the network of materials serves as cages to retain the EVs. The caging process has to be completed before the formation of the meshes, meaning the EVs are mixed directly with the raw material to form a mixture, followed by the curing of hydrogels or the setting of inorganic mineral bulks to establish the meshes. This has been demonstrated by the procedures of preparation in many bulk EV delivery systems^[52,53,61,105]^.

The major mode for EVs to leave bulk materials is diffusion, which is controlled by the degradation of the bulk material. Diffusion is driven by the concentration (or the chemical potential) of EVs. The initial concentration of EVs, often in the range of 10^9^ to 10^12^ particles/mL, is the highest, standing for maximum potential for EVs to diffuse out of the material. This is followed by a sustained release close to a constant rate (zeroth-order), but as time passes, the concentration lowers, so that the rate of diffusion is gradually reduced and ultimately tends to zero. 

**Figure 2:**
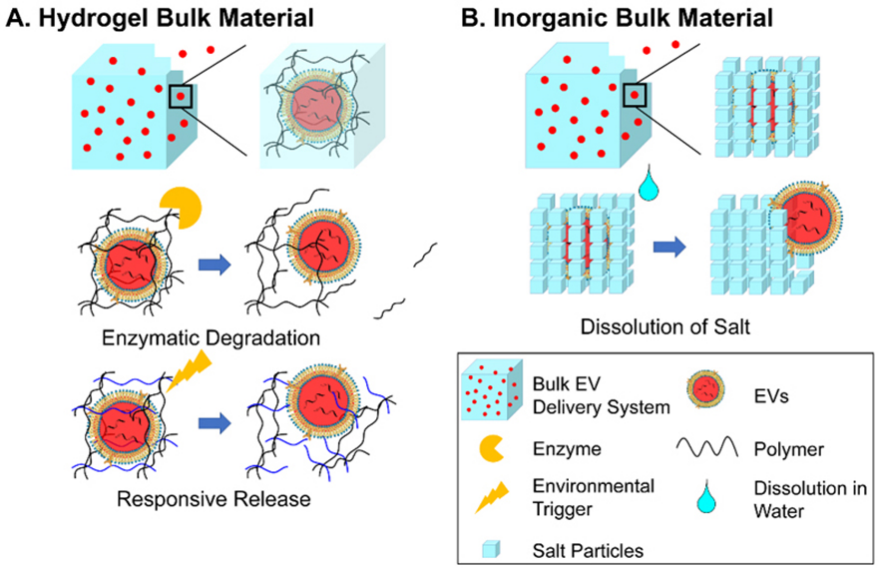
Schematic illustration of the mode of action of bulk EV delivery systems through EV-encapsulation and release by degradation: (A) Enzymatic degradation and responsive release by hydrogels and (B) release by dissolution of inorganic bulk materials. EVs: Extracellular vesicles.

In addition, the existence of meshes prolongs the diffusion of EVs for bulk materials. The average size of the meshes is generally considered smaller than the diameter of the encapsulated EVs, while a multi-layered mesh structure is also present to increase the number of meshes for EVs to diffuse through. These two structural parameters are the factors in retaining EVs. The enlargement of meshes and the decrease in mesh numbers mark the lifting of the restrictions and the start of the release of EV. Such a change in the mesh is mainly caused by the passive degradation of the material. For water-soluble polymers, degradation occurs through biodegradation by enzymes secreted by the coming cells. Due to the smaller size of cells compared to the meshes, the biodegradation of hydrogel starts from the surface of the hydrogel and then moves inward, which is an accelerating procedure with more availability of enzymes from the increasing number of attached cells and greater infiltration of enzymes into the looser and decomposed hydrogel^[119]^, until the final remnants of hydrogels with little surface available for enzymatic reaction [Figure 2A]. For inorganic bulks, dissolution (inorganic salts) of the network is the major process limiting the diffusion of EVs [Figure 2B]. However, to the best of our knowledge, there is no reported comparison between the release of EVs versus the dissolution of salt^[113]^. It can be speculated that the process takes a similar curve as the biodegradation of the hydrogel. As water gradually breaks the intermolecular or interionic bonds, water molecules infiltrate more easily through the new defects from the dissolved parts until full dissolution of the salt.

The relative relationship between the rates of EV diffusion and hydrogel degradation determines the overall release profile *in vitro*, which can be measured by quantification of specific antibodies^[52]^, particle number^[53]^ and the intensity of fluorescence labels^[61]^ of the released EVs after specific lengths of time. At the starting stage (0-~30% accumulative release of EVs), the high potential of EVs may cause a burst release of EVs, as observed in some reported delivery systems^[74,120]^. As the delivery proceeds into the middle stage (~30%-~80% cumulative release of EVs), the reduced potential of EVs and the accelerated degradation cancel out, reaching a close-to-constant release of EVs, which is the key stage of bulk material delivery systems. This stage can cover three^[119]^ to more than 30 days^[62,121]^, depending on the material structures and the need for recovery of the injury. A well-tuned hydrogel can even fully match the release profile of EVs with the degradation profile, as illustrated by almost identical curves of material degradation and EV release in Zhang and colleagues’ HAMA hydrogel scaffold^[53]^. In the final stage, the chemical potential of EVs is too low to overcome the obstacle of diffusion from the remaining hydrogel, leading to non-100% release until the full degradation of the hydrogel. 

**Figure 3:**
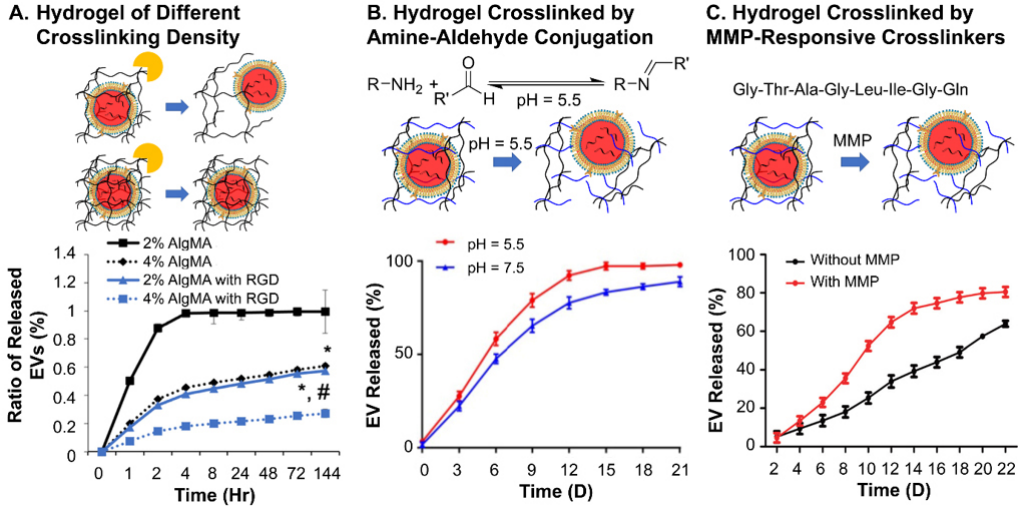
*In vitro* EV release profiles of (A) Hydrogels without responsiveness of different crosslinking densities; (B) acid-responsive hydrogels crosslinked by amine-aldehyde conjugation; (C) enzyme-responsive hydrogels. Figure 3A adapted with permission^[105]^, Copyright 2021, Elsevier; 3B adapted with permission under terms of the CC-BY NC license^[63]^, Copyright 2019, the authors, published by Ivyspring International; 3C adapted with permission^[122]^, Copyright 2019, Royal Society of Chemistry. EV: Extracellular vesicle.

At the macroscopic level of material, a high crosslinking density can both reduce the mesh size and add the number of meshes in the material, and at the same time slow down the degradation rate. This can effectively extend the retainment of EVs, as demonstrated by the faster release (~90% over 2 h) from 2% AlgMA hydrogel than that (~40% over 2 h) of 4% AlgMA hydrogel in the study by Huang and coauthors [Figure 3A]^[105]^. Some hydrogel delivery systems incorporated stimuli-responsive crosslinking. Aldehyde-amine crosslinking is sensitive to an acidic environment. Wang *et al.* showed the difference in EV release under acidic pH of 5.5 compared to that at 7.5 in the injectable hydrogel of oxidized hyaluronic acid and poly-*ε*-L-lysine^[63]^. The hydrogel showed slightly faster and more thorough release in an acidic environment (~80% at pH 5.5 compared to ~65% at pH 7.5 on Day 12) [Figure 3B]. Similar results were reported by Jiang *et al.* in their injectable phenylboronic acid-modified hyaluronic acid-PVA hydrogel, which showed faster release of EVs from neural stem cells after treatment with glucose and/or H_2_O_2_^[68]^. Some hydrogels incorporate enzymatically responsive crosslinkers, such as matrix metalloprotease cleavable polypeptide (Gly-Thr-Ala-Gly-Leu-Ile-Gly-Gln), as reported by Han *et al.*^[122]^. Due to the relatively high crosslinking density, an obvious accelerated release of EVs has been observed [Figure 3C]. The enzyme-responsive hydrogel released ~70% EVs over 14 days followed by the plateau of release at 80%, while the non-responsive hydrogel followed a relatively linear profile to the delivery of 60% of EVs over 22 days. Microscopically, a homogeneous distribution of crosslinking points within the hydrogel is also crucial^[123]^, which has not been reported in EV delivery systems but frequently in conventional drug delivery systems. Although considered smaller than that of EVs, the mesh size has a large deviation within a bulk hydrogel, leading to inconsistent degradation, incomplete encapsulation and inconstant release profile of the material, especially for hydrogels crosslinked by reversible crosslinking moieties, which do not possess homogeneously distributed and stable crosslinking structures^[62,120,123,124]^. Such a non-uniform crosslinking system also suggests the heterogeneous distribution of EVs within the hydrogel, especially exposed at the surface of the material. These molecular defects all contribute to the possible burst release at the start of the delivery of hydrogel EV delivery systems. Similarly, for bulk inorganic materials, thorough mixing of the salt and EVs is necessary for the constant release of EVs. 

In addition to the crosslinking structures and the distribution of EVs in the material, other parameters can also affect the diffusion of EVs from hydrogels. The change of stiffness of physically crosslinked hydrogel (i.e., stress-relaxing hydrogels) is one of such kinds. In their study on the impact of stiffness of hydrogel on the release of EVs, Lenzini and colleagues adjusted the Ca^2+^ concentration to achieve soft (shear modulus ~500 Pa) and stiff (shear modulus ~3 kPa) alginate hydrogel without a dramatic change in mesh size^[70]^. It was found that EVs could still diffuse through a stiff Ca^2+^-crosslinked alginate hydrogel compared to softer or non-stress-relaxing hydrogels. This indicated the possible diffusion by the deformity of EVs under the confinement of stiff stress-relaxing hydrogels. Such a fact also suggests that flexible polymer chains in hydrogel may also affect the diffusion of EVs. A hydrogel of polymers with low *T_g_*, such as polyethyleneglycol (*T_g_* around -63 °C^[125]^), is supposedly softer compared to hydrogels of polymers with high *T_g_*, such as hyaluronic acid, given the same crosslinking density. However, no such study has been made to the best of our knowledge.

**Figure 4:**
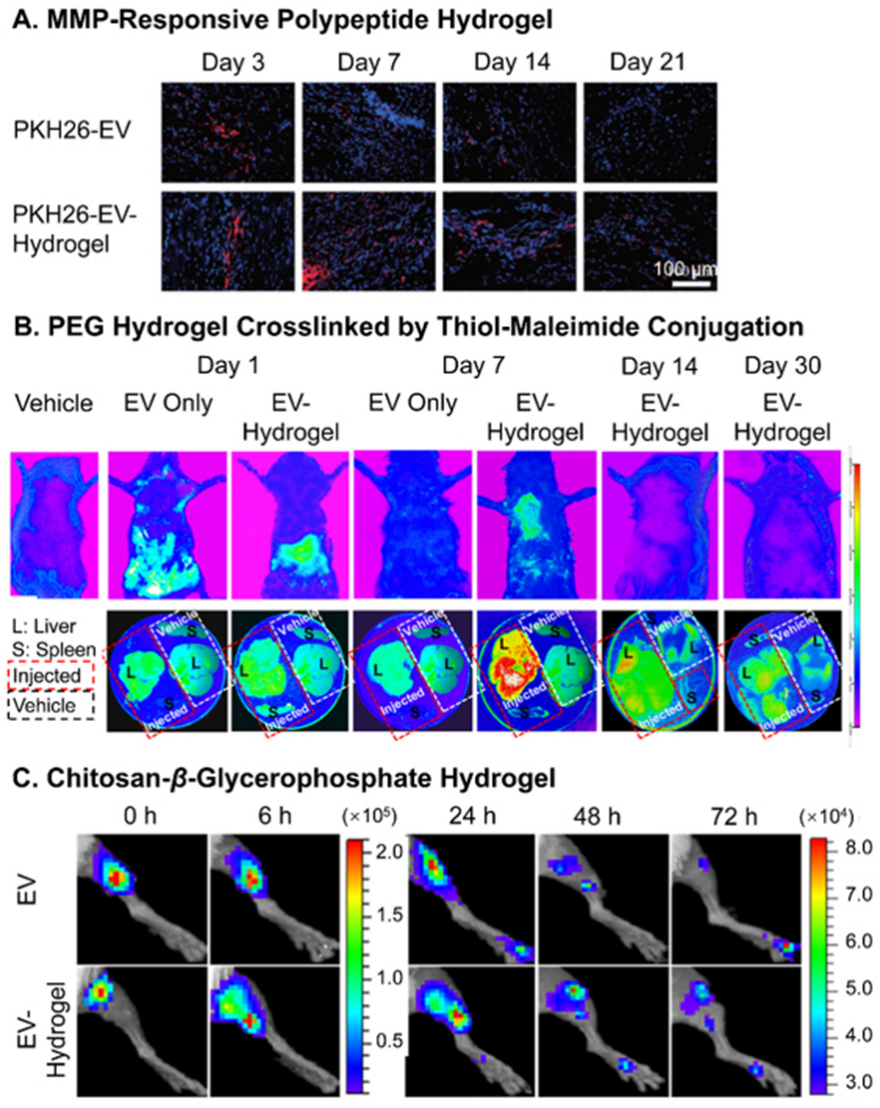
*In vivo* tracking of EVs released from hydrogels. (A) Fluorescence microscopy of PKH-26 red-labeled hUCMSC-EVs released by the MMP-responsive hydrogel in the border region of myocardial infarction; (B) Live and *ex vivo* imaging of PKH-26 red-labeled EV delivery PEG hydrogels crosslinked by thiol-maleimide conjugation in the chronic liver injury model; (C) Live imaging of *Gaussia* luciferase-labeled MSC-EVs encapsulated in chitosan-*β*-glycerophosphate hydrogel injected in ischemic hindlimb. Figure 4A adapted with permission^[122]^, Copyright 2019, Royal Society of Chemistry; 4B adapted with permission^[62]^, Copyright 2019, American Chemical Society; 4C adapted with permission^[126]^, Copyright 2018, American Chemical Society. EVs: Extracellular vesicles; PEG: poly(ethylene glycol); MSC: mesenchymal stem cell.

The tracking of *in vivo* release profiles is a lot more complicated compared to *in vitro* observations, which makes such information highly limited. The major approach to observing the traces of EVs *in vivo* is through qualitative or semiquantitative fluorescence signals of labeled EVs. Han *et al.* tracked the PKH-26 red signal of hUCMSC-EVs released by the enzyme-responsive hydrogels mentioned above in the border region of myocardial infarction^[122]^. The retention of EVs matched the *in vitro* release profile with discernable existence at day 21 in the tissue, while no trace of EVs was found for injections with sole EVs [Figure 4A]. Such labeled EVs can also be observed by live and *ex vivo* bioluminescence imaging to locate their distribution after release. Mardpour and colleagues encapsulated MSC-EVs in thiol-maleimide PEG hydrogels to treat chronicle liver failure^[62]^. The live and *ex vivo* imaging clearly showed maximum retention of released EVs in liver after seven days and gradual decrease till Day 30, while no trace was found for EV-only groups at the same time spots [Figure 4B]. Similarly, Zhang *et al.* presented the biodistribution of *Gaussia* luciferase labeled MSC-EVs encapsulated in amine-aldehyde chitosan hydrogels for blood vessel regeneration of ischemic hindlimbs [Figure 4C]^[126]^. In combination with quantitative analysis results of fluorescence intensity and component (miRNA-126), the hydrogel successfully retained the EVs within the hindlimb for over 72 h. 

From the cases above, it can be seen that the bulk materials have been extensively applied to achieve the successful retainment of EVs. Especially for hydrogels, their release profile can be adjusted primarily through the crosslinking strategies and the crosslinking density. However, more structural parameters, such as the intrinsic flexibility of the polymers and the homogeneity of crosslinking density, can be investigated for their impact on the release performance of hydrogels. Moreover, the different delivery performance *in vivo* of responsive crosslinking designs is difficult to reflect in current studies. On the other hand, limited by the rare studies of inorganic bulk material, the correlation of material structure, properties and release performance is still unclear.

### For scaffold materials: two different modes

**Figure 5:**
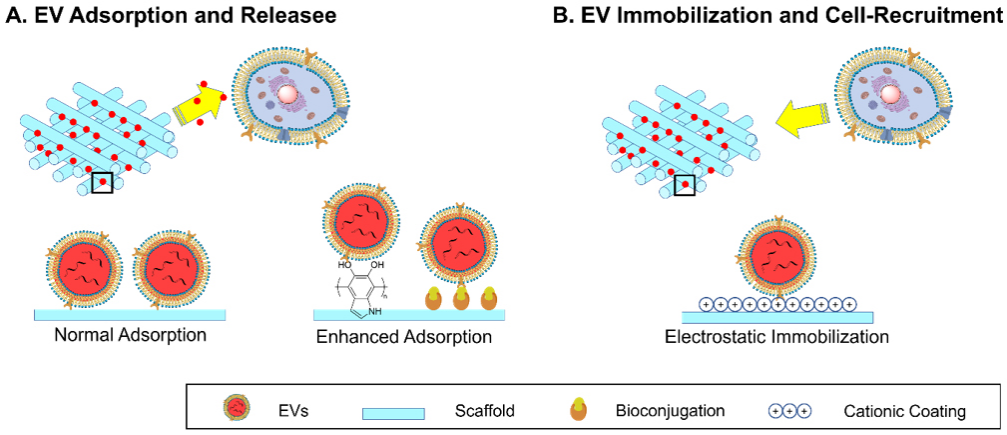
Schematic illustration of two modes of action of EV delivery scaffolds: (A) Surface adsorption and release of EVs and (B) surface immobilization of EVs and cell recruitment. EVs: Extracellular vesicles.

As mentioned in “DESIGN STRATEGIES OF MATERIALS FOR EV DELIVERY SYSTEMS”, most EV delivery scaffolds have EVs attached. Depending on the status of EV attachment, the delivery mechanism can be divided into two modes [Figure 5].

#### EV-adsorption and release

The first EV attaching mode is through adsorption to scaffolds, and accordingly, the delivery is through the desorption and release. Equation 1 depicts the reversible relationship between adsorption and desorption:

**Figure:**
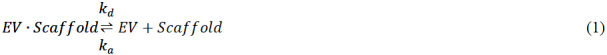


where *EV · Scaffold *denotes the EV attached state and *EV + Scaffold *means EVs are released. *k_d_* stands for the coefficient of desorption and *k_a_* for adsorption. The adsorption-desorption is a reversible process. *k_d_* is affected by multiple factors, such as the concentration difference of EVs between the scaffold and the environment and the liquid shear force within the injury, which can be treated as a constant here. *k_a_* is dependent on the interactions of EVs and scaffold surface, and also the concentration difference of EVs.

**Figure 6:**
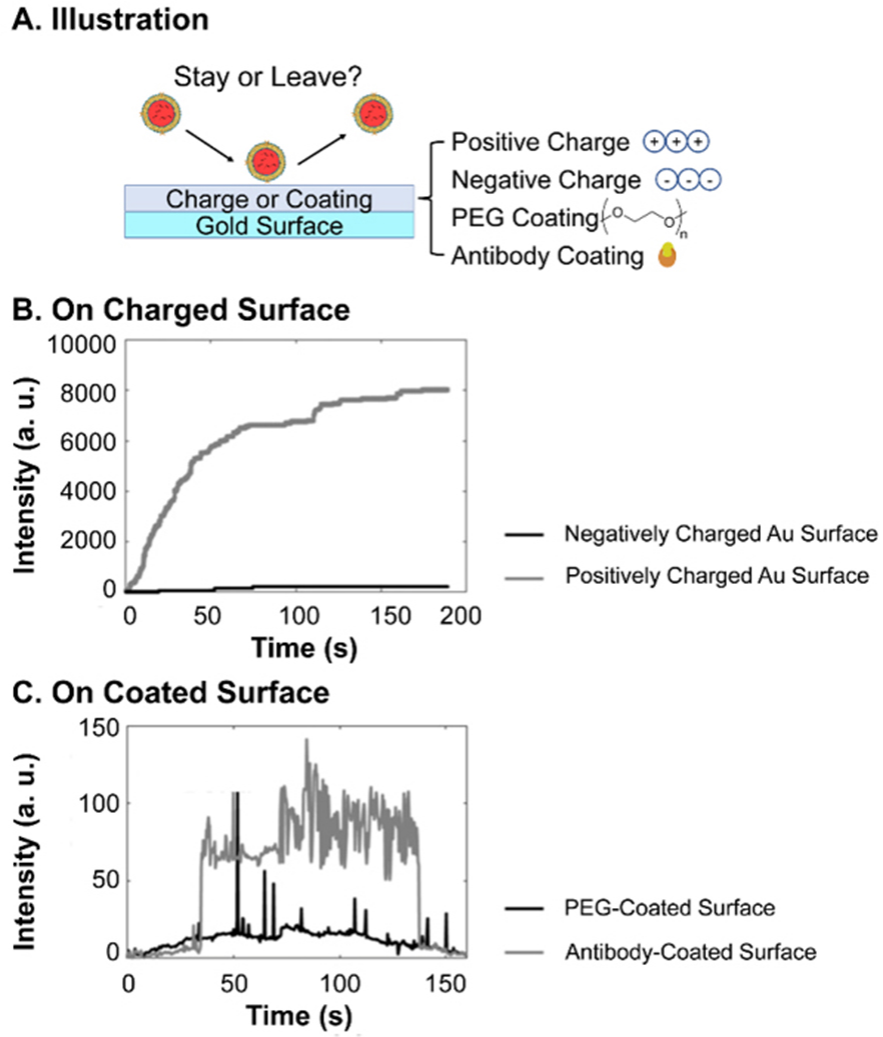
Interferometric plasmonic microscopy detection of the number of EVs from A549 cell lines binding different surfaces: (A) Illustration of the experiment; (B) EVs binding positive and negatively charged gold surfaces. Positively charged surface shows immobilization effect while negatively charged surface shows bouncing off of EVs; (C) EVs binding PEG and antibody-coated gold Surface. PEG surface shows bouncing off of EVs while antibody-coated surface shows an “adsorb-stay-desorb” procedure. Adaptations with permission^[130]^, Copyright 2018, National Academy of Sciences. EVs: Extracellular vesicles; PEG: poly(ethylene glycol).

In the preparation of most EV delivery scaffolds, the incubation of scaffolds with EV suspensions is the basic method to load EVs. A higher concentration of EVs can lead to a higher *k_a_* and thus a larger loading efficiency. However, when transplanted to tissue, *k_a_* significantly decreases due to almost zero presence of EVs of the same type in injured tissue. Therefore, adsorption alone is unstable and a burst release can be expected from mineral scaffolds or non-treated polymeric scaffolds^[109,127,128]^. An increment of *k_a_* by enhancing the EV-surface interaction is necessary for a more controlled release profile. Thus, the adsorption and desorption of EVs to different surfaces of material are crucial to their loading and release. Although previous reports have provided semiquantitative characterizations of the coverage of EVs adsorbed on scaffolds using fluorescence microscopy^[97]^ or electron microscopy^[129]^ as a result of adsorption, followed by the release profile of the EVs as a result of desorption, rarely direct investigations have been made, possibly due to the difficulties in the detection of nanosized EVs with sufficient resolution of the whole process^[130]^. The only research, to the best of our knowledge, was conducted by Yang and colleagues^[130]^. They developed interferometric plasmonic microscopy for imaging and size analysis of the adsorption and desorption of exosomes to negatively charged gold, positively charged gold, PEG-modified gold and CD63 antibody-modified gold surfaces. For the different charges of the surfaces, positive charge exhibited the strongest attraction of EVs from A549 cell lines, i.e., highest *k_a_*, to adsorb to the surfaces, compared to few (*k_a_* ≈ 0) on negatively charged ones [Figure 6]. On surfaces with different chemical properties, EVs bounced off PEG-modified gold surfaces, as a representative superhydrophilic material with reverse-osmotic properties, while an obvious “adsorb-stay-desorb” procedure was observed for CD63 modified surfaces. This is due to the weak but insufficient interaction, or insufficient *k_a_*, between the antibody and the EVs. The results suggest that in order to promote the loading efficiency and ensure more sustained release, a properly strong interaction and *k_a_* between the EVs and surfaces of scaffolds are required.

EVs have phospholipid bilayer membrane structures with proteins and peptidoglycan attached to or inserted within. The phosphates groups on phospholipids, and amide groups, amine groups, hydroxy groups and carboxylic groups on the proteins and peptidoglycans are able to interact with the scaffolds by hydrogen bonds or van der Waals forces when polar carbonyl groups are present, such as PCL, PLLA and PLGA. This is the case when the surface of the materials is not modified. 

**Figure 7:**
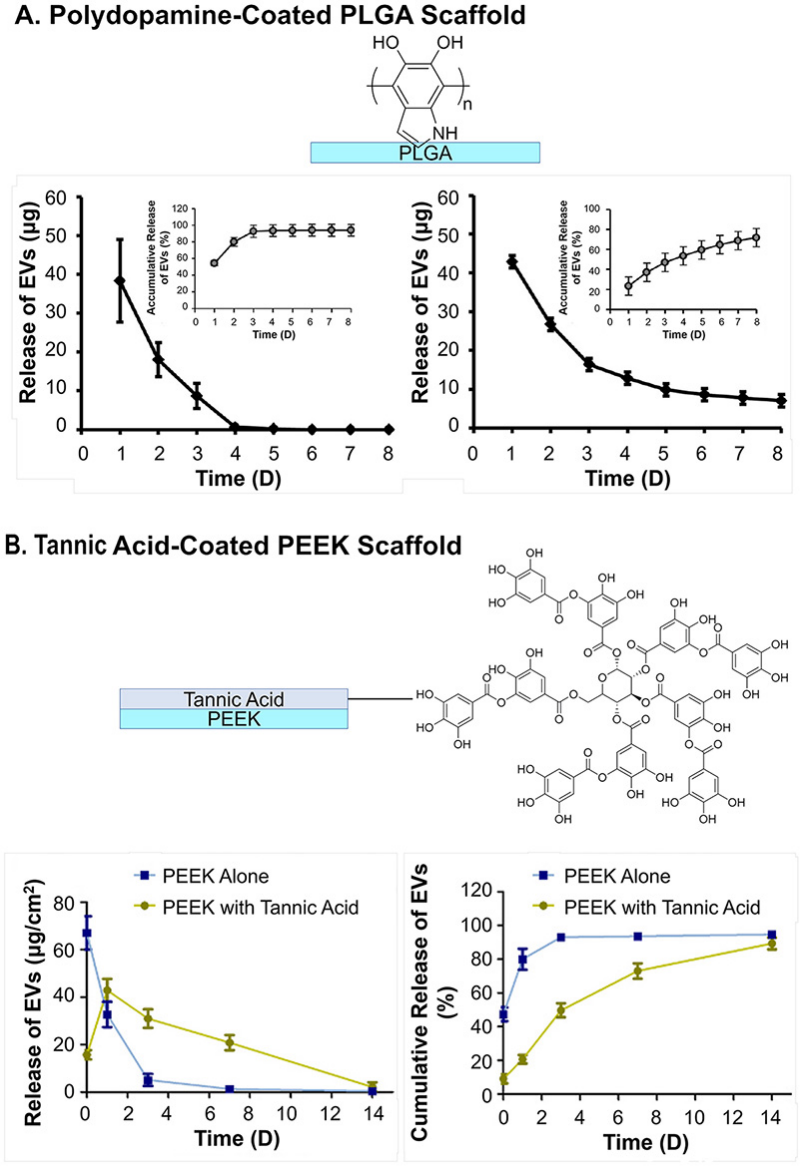
The *in vitro* release profile of EVs from scaffolds prolonged by coatings: (A) Polydopamine-coated PLGA scaffold and (B) tannic acid-coated PEEK scaffold. Figure 7A adapted with permission^[97]^, Copyright 2018, American Chemical Society; 7B is adapted with permission^[101]^, Copyright 2021, the authors, published by KeAi Publishing. EVs: Extracellular vesicles; PLGA: poly(lactic-*co*-glycolic acid).

**Figure 8:**
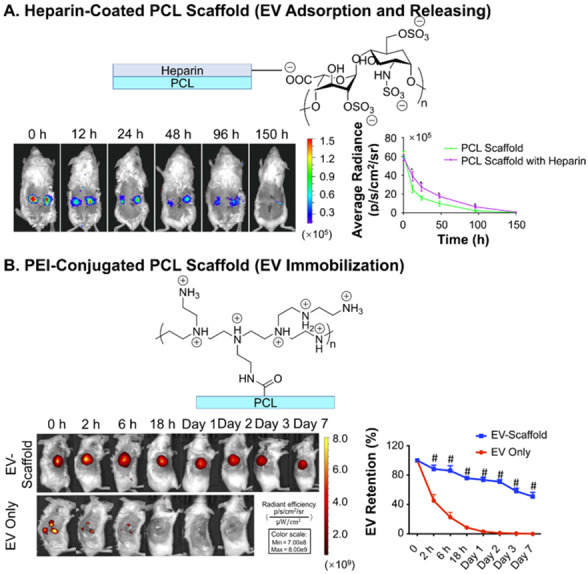
*In vivo* tracking of EVs delivered from scaffolds. (A) Live imaging of the retained delivery of Luciferase-labeled EVs by PCL scaffold coated with heparin at abdominal artery of mice; (B) live imaging of the immobilized EVs on PCL scaffold conjugated with PEI implanted on large skin wound of mice. Figure 8A adapted with permission^[93]^, Copyright 2019, Elsevier; 8B adapted with permission under terms of the CC-BY NC license^[92]^, Copyright 2021, the authors, published by American Association for the Advancement of Science. EVs: Extracellular vesicles; PCL: polycaprolactone; PEI: polyethyleneimine.

One way of enhancing adsorption is to raise the density of hydrogen bonds or van der Waals forces between EVs and scaffold surfaces. Polydopamine coating is the most applied method to increase the *k_a_*. Inspired by the adsorption of mussels on solid surfaces, researchers have applied polydopamines to introduce a high number of hydrogen bond donors (hydroxy on catechol groups) and exert π-π interactions (benzene of catechol groups) between surfaces^[131]^. In the report on poly(lactic-*co*-glycolic acid) (PLGA) EV delivery scaffolds for bone regeneration, Li *et al.* showed that the coating of polydopamine can significantly reduce the burst release of EVs from ~60% to ~20% of the total load, and retention from three days to more than eight days^[97]^. Since the membrane of EVs is mainly composed of phospholipids, they are hydrogen bond acceptors as well as negatively charged under physiological conditions, and thus can be adsorbed with polydopamine coating with a higher probability of extending the release [Figure 7A]^[97]^. Tannic acid, with a similar catechol structure as polydopamine, has also been used. Under oxidizing and basic conditions, the catechol structures of tannic acid allow it to self-polymerize and become adhesive reminiscent of polydopamine, as demonstrated by the PEEK scaffolds modified by Fan *et al.*^[101]^. With the coating of tannic acid, the surface hydrophilicity (i.e., water contact angles ranging from 60 ° to 30 ° after coating) was greatly promoted. Accordingly, an obvious reduction in burst release (50% to 10% release at Day 0 after coating) and more linear release (three days to 14 days to reach 90% release after coating) were both observed [Figure 7B]. Specific bioconjugation has also been exploited to mimic natural EV-ECM complex structures^[132]^ by applying a coating of ECM involved polymers. Heparin has been used to anchor biomolecules such as growth factors, and was recently also applied as coating of scaffolds by Wei *et al* to treat hyperlipidemia^[93]^. A heparin coating on a polycaprolactone (PCL) scaffold for blood vessel graft was implanted on the abdominal aorta. The coating slightly slowed down the release of the vesicles on the scaffold, as shown by the *in vivo* fluorescence live imaging of luciferase labeled EVs [Figure 8A]^[93]^. This is one of the few reported *in vivo* EV release behavior of scaffolds. Similarly, the bioconjugation effect has also been reflected by the prolonged release profiles of scaffolds made from or incorporated with decellularized ECM materials, including collagen (over 14 days release)^[133]^ and decellularized cartilage (more than 50% compared to blank, 14 days *in vitro* release)^[104]^. Another approach for increasing hydrogen bonds is through the modification of the surfaces of scaffolds, exploiting antibodies specific to the antigens on EVs, as was shown by the CD63-A549 cell line-derived EVs in the interferometric plasmonic microscopy test by Yang *et al*.^[130]^. Another example is the integrin α4β1 ligand LLP2A-placenta mesenchymal stem cell-derived EVs on electrospun PLLA-PCL scaffolds by Hao *et al.*, but no information was given on the release profile of the EVs^[134]^.

Compared to weak hydrogen bonds and van der Waals interaction, electrostatic interactions are much stronger and more effective in retaining EVs. The majority of phosphate groups on the membrane of EVs make them overall negatively charged. Some scaffolds with relatively weak cationic charge can also extend the release of EVs by increasing *k_a_* through electrostatic interactions, such as chitosan (80% in six days *in vitro*)^[90]^.

#### EV-immobilization and cell-recruitment

The fixed mode of EV attachment is less frequently utilized compared to the release mode. It involves the full immobilization of EVs on the surfaces of scaffolds for the recruitment of targeted cells instead of releasing them. In this case, *k_a_* is much larger than *k_d_*, so the overall reaction is one way to reach the *EV · Scaffold *state. As presented by Yang and colleagues, positively charged coatings are ideal for achieving this purpose^[130]^. PEI coated upon scaffolds or conjugated to carboxylic moieties to the scaffolds can greatly promote the cationic charge on the material. This can lead to strong electrostatic attraction and subsequent full immobilization of negatively charged EVs. Su and coauthors covalently conjugated PEI to an electrospun PCL scaffold to acquire a high positive charge density^[92]^. The material could thus immobilize 75% of the supplied mesenchymal stromal exosomes compared to almost none on the untreated scaffold without further release until consumed by attached cells. The scaffold thus acted as the recruiter and trainer for macrophage and T cells to facilitate skin recovery. Of course, due to the hydrolysis of PCL, the uninternalized EVs were eventually released. The retention rate was 50% after seven days *in vivo* compared to the two-day elimination without material based on the quantitative analysis of fluorescence intensity [Figure 8B].

It follows that surface modifications with specific interactions with EVs have shown to greatly increase the retainment of adsorbed EVs on scaffolds, both *in vitro* and *in vivo*. This has been the mainstream for the preparation of EV delivery systems in scaffold forms. However, a deeper understanding of the intensities of the interaction between the coatings and EVs is necessary for better control of the delivery of EVs. Meanwhile, porosity, the key feature of scaffolds, has not been mentioned in any of the above-discussed studies. This parameter affects the overall loading capacity of EVs and should receive more emphasis in further investigations.

### Promotion of tissue regeneration by EV delivery systems

Despite the need for both an in-depth understanding of the interaction between the delivery system and the release of EVs, and more accurate control over stimuli-responsive release, both bulk and scaffold materials have shown good prolonged EV delivery compared to the administration of only EVs and the results are obvious in the regeneration of tissue, as reflected by the many reports of their use in treating various injuries at different sites of the body. 

**Figure 9:**
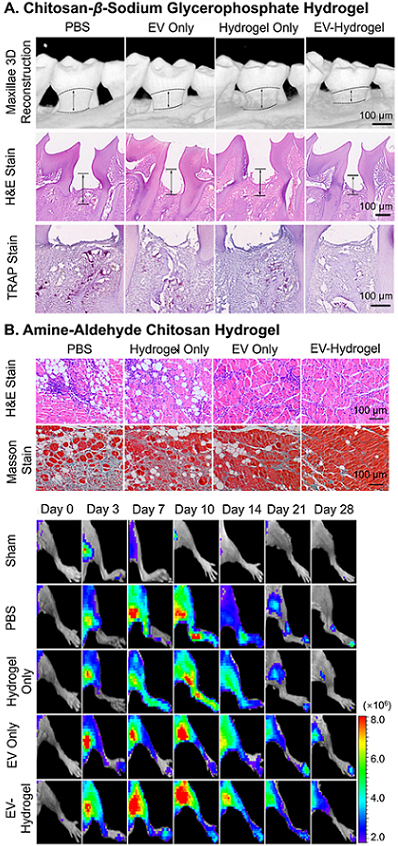
Recovery results of EV delivery hydrogels. (A) Accelerated healing of alveolar bone by dental pulp stem cell-EV chitosan-*β*-sodium glycerophosphate hydrogels injected within the cavity of periodontitis; (B) reduction of necrotic fibers and inflammatory cells (staining), and improved angiogenesis (live imaging) by MSC-EV-encapsulated amine-aldehyde chitosan hydrogel in hindlimb. Figure 9A adapted with permission^[47]^, Copyright 2020, the authors, published by KeAi Publishing; 9B adapted with permission^[126]^, Copyright 2018, American Chemical Society. EV: Extracellular vesicle; MSC: mesenchymal stem cell.

Concerning bulk materials to treat periodontitis, Shen and colleagues injected chitosan-*β*-sodium glycerophosphate hydrogel encapsulating dental pulp stem cell-derived EVs into the cavity of periodontitis^[47]^. Compared to the EV-only group, the groups injected with EV-hydrogel showed an approximately 1.5-time acceleration of the healing of alveolar bone [Figure 9A] and a two-time reduction in the loss of bone, which also matched the reduced amount of cytokine and the increased number of macrophages in anti-inflammatory phenotype for the EV delivery system. In the field of cardiac repair, hUCMSC EVs in functional MMP-responsive polypeptide hydrogels reported by Han and coauthors exhibited an approximately 50% reduction in inflammation, fibrosis and apoptosis, and upregulated angiogenesis for myocardial infarction 28 days after surgery compared to EV-only treatment, as a result of obvious retention of EVs [Figure 4A]^[122]^. A similar result was observed in ischemic limbs parallelly treated with MSC-EVs and MSC-EV-chitosan hydrogel, as shown in Figure 4C^[126]^. The localized retention of EVs by the material directly led to the reduction of necrotic fibers and inflammatory cells, and improved angiogenesis at both cellular and physiological levels, resulting in optimal muscle recovery after 14-28 days [Figure 9B]. More cases have highlighted the regenerative promise of the administration of EV delivery systems compared to EVs alone^[51,56,74,135]^.

**Figure 10:**
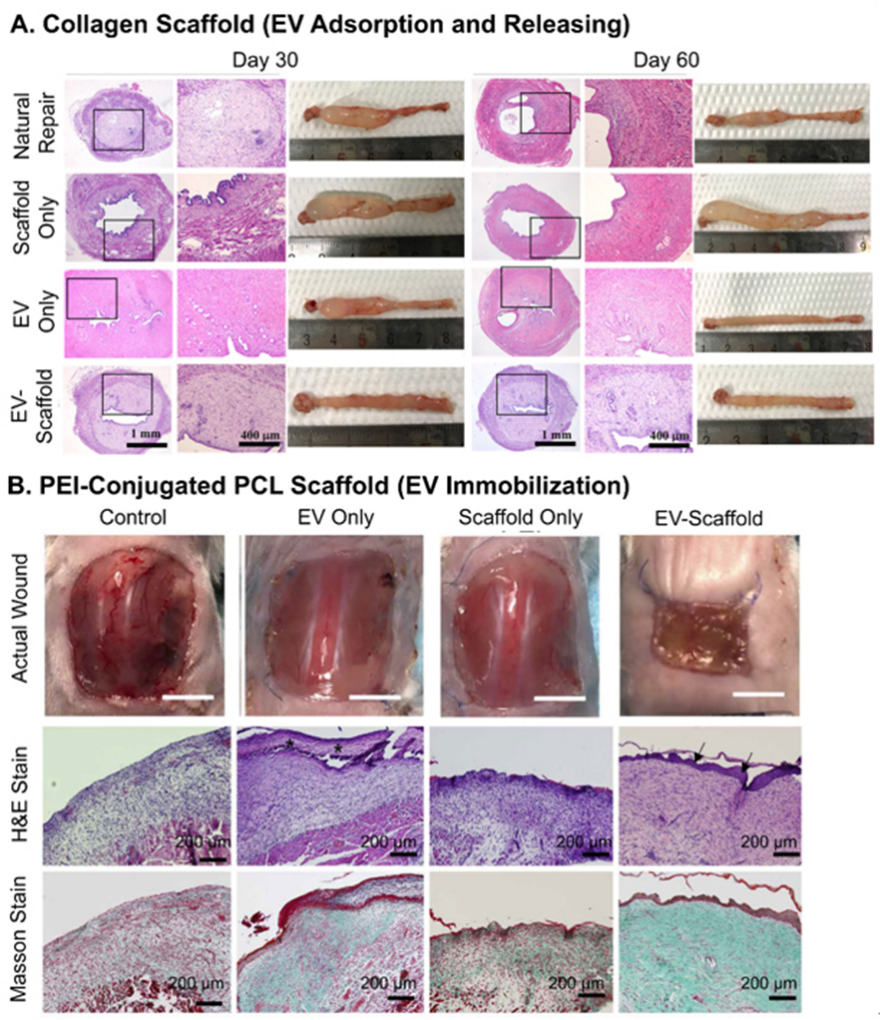
Recovery results of EV delivery scaffolds. (A) Promoted regeneration of glands and reduction of muscular dystrophy by hUCMSC-EVs laden collagen scaffolds for endometrium injury; (B) faster wound closing (picture) and more formation of new epidermis (staining) by MSC-EVs immobilized on PEI-conjugated PCL scaffolds treating large skin wound. Figure 10A adapted with permission^[133]^, Copyright 2020, Elsevier; 10B adapted with permission under terms of the CC-BY NC license^[92]^, Copyright 2021, the authors, published by American Association for the Advancement of Science. EV: Extracellular vesicle; MSC: mesenchymal stem cell.

As for scaffolds, not as many reports compared the results of EV-only and EV-scaffolds groups, since EVs are mostly considered as an enhancement to the functions of scaffolds. Answering the needs for the regeneration of endometrium and fertility, Xin and colleagues loaded hUCMSC-EVs to collagen scaffolds, which could sustain the release over 14 days in an extended burst release manner^[133]^. The combination of EVs and scaffolds was found to achieve the results of EV-only group in terms of regenerated endometrium thickness and the number of PR^+^ cells, and have improved effects on the regeneration of glands and reduction of muscular dystrophy after 30 and 60 days of administration [Figure 10A]. Jiang *et al.* exploited acellular ECM scaffolds laden with Wharton’s jelly MSC-EVs to induce osteochondral regeneration^[82]^. The EV-scaffolds group was assessed to have promoted ~33% of the recovery of cartilage compared to EV-only group three and six months after transplantation. In the representative case of cell-recruiting mode, the PCL scaffolds immobilized with MSC-EVs through conjugated PEI were used to treat square skin excisional wounds with an area of 225 mm^2[92]^. After transplantation for two weeks, a lot more newly formed epidermis and the presence of *α*-smooth muscle actin were found in the tissue using the combinatory material. The EV-scaffolds group exhibited strong function, reaching ~70% of wound closure, while the closure of EV-only group was only 30% and scaffold-only group 20% [Figure 10B].

**Figure 11:**
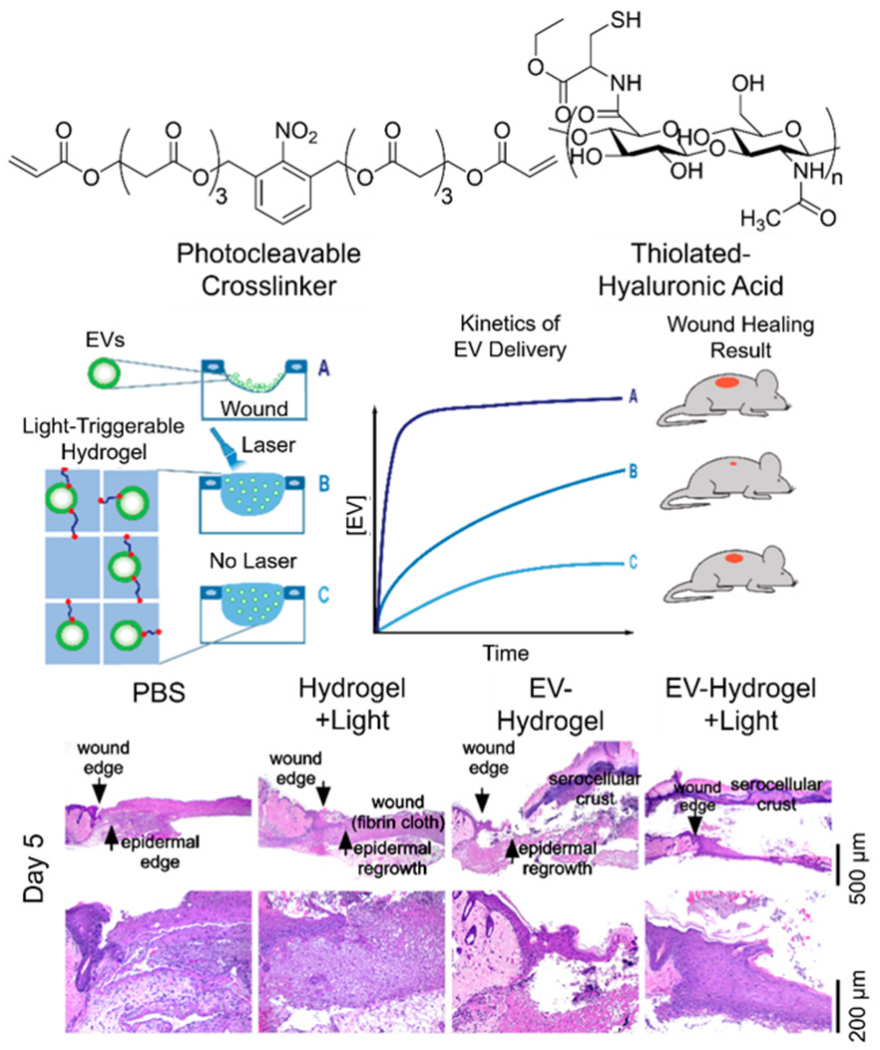
Schematic illustration of the promoted wound-healing effect of controlled release of EVs from photocleavable hydrogel and the according staining of wounds treated with different approaches at Day five. Adaptations with permission^[136]^, Copyright 2019, American Chemical Society. EVs: Extracellular vesicles.

With greatly promoted regenerative outcomes resulted from EV delivery systems, the followed key question is whether the *in vivo *kinetics of delivery agrees with the physiological patterns of stem cells, which is crucial for the regeneration of tissue and the closing of injuries. The tracking of the internalization of EVs delivered by the bulk materials is thus necessary for the recovery procedure of tissue. This is illustrated by the study conducted by Henriques-Antunes *et al.* on the relationship between the EV release profile and the healing of diabetic chronic wounds^[136]^. In order to achieve a fully controlled release of EVs in terms of dose and frequency, a photocleavable crosslinker was synthesized to conjugate EVs and thiolated hyaluronic acid to form a hydrogel, so that the delivery can be proceeded by periodical irradiation. It was found that the matching of the release profile of EVs and the dynamics of the regeneration of skin can promote more than 40% healing effect compared to scaffold alone and single administration of EVs [Figure 11]. This single study calls for more investigations based on different kinetics of EV release and the interaction with cells from different tissues. 

## ADDITIONAL FUNCTIONS OF EV DELIVERY SYSTEMS

In order to enhance the EV delivery systems to be more suitable for specific conditions in diseases or requirements of surgeries, extra functions have been incorporated into the materials through special processing strategies based on the intrinsic properties of the materials or the introduction of specific functional groups/substances to change the property of the material at the molecular level.

In many cases, minimally invasive surgery is required to reduce the suffering of patients. Injectable hydrogels have met such requirements through shear-thinning properties or mixing with double-barrel syringes, but for materials such as scaffolds and photocrosslinkable hydrogels, transplantation is still needed. To compensate for this, the size of these delivery systems must be sufficiently small, i.e., in micrometer class, for direct injection. So far, the primary approach to miniaturizing EV delivery systems is through the formation of emulsion. To fabricate hydrogel microspheres, water-in-oil emulsion is used. This is demonstrated by Liu *et al.*, who developed chitosan microspheres after high-speed homogenization of acidified chitosan-liquid paraffin/petroleum ether/SPAN80 emulsion system^[137]^. With a diameter of 5 μm, the cationic charged spheres were incubated with anionic rabies virus glycoprotein peptide-modified EVs loaded with reactive oxygen species-responsive curcumin nanoparticles. Although the microspheres were prepared only for better observation of EVs, it suggests the possibility of using microspheres as EV carriers through adsorption and such a concept can be extended in future exploration of EV-based therapies. As for the fabrication of non-water-soluble scaffolds, reverse phase emulsion or double emulsion systems can be applied. Hollow PLGA spheres with a diameter of 50 μm were prepared using water-in-oil-in-water double emulsion systems with the help of sonication. The spheres were eventually surface modified with polydopamine for the attachment of serum-derived EVs^[138]^. The EV-loaded microspheres were shown to be internalized by DC2.4 and RAW264.7 cells, with the internalization promoted by higher EV loading rates. Such a double emulsion system also allows the use of microfluidic devices for the fabrication of microspheres with more uniform sizes. Through a well-designed PDMS microfluidic chip, Swanson and colleagues were able to fabricate human DPSC-derived EV-encapsulated microspheres with a diameter of around 5 μm, which were later physically adsorbed to poly(L-lactide) (PLLA) scaffolds and transplanted to treat defected calvary of mice^[127]^. Such a double scaffold system utilized a two-step release, i.e., desorption of microspheres from scaffold and hydrolysis of microsphere to release EVs. The system was found to make the delivery of EVs more linear. It has to be noted that in order not to disrupt the encapsulated EVs by organic solvent, the oil phases of the above-reported spheres were volatile for thorough evaporation and easy collection. Accordingly, dichloromethane and ethyl acetate were used in the above-mentioned two studies. However, the remnant organic solvent is still potentially harmful to tissue and EVs. A replacement of oil phase with air and *in situ* crosslinking can allow the direct formation of hydrogel microspheres encapsulating EVs within the injured tissue, without the need to remove the organic solvent, as demonstrated by Yao and colleagues^[139]^. They filled a double-barrel syringe with fibrinogen/EVs mixture as the polymer solution and thrombin solution as the crosslinker, and equipped the tip of the syringe with a spray needle. By spraying out the combined solution, the droplets crosslinked *in situ* and were sent directly at the myocardial infarction area of a pig model to achieve the purpose of minimum invasive surgery.

Oxidative stress is another frequent problem for tissue regeneration due to a lack of oxygen. Localized supply of oxygen combined with the removal of reactive oxygen species can enhance the result of treatment in addition to the delivery of EVs, especially for chronic diabetic patients. Shiekh and coauthors designed a special polyurethane scaffold for this purpose^[99]^. The polyurethane was specifically designed to be an antioxidant with vitamin C as one of the monomers to as scavenger of reactive oxygen species [Table 2], while the authors also doped calcium peroxide, which can decompose and produce oxygen when in contact with water, into the polyurethane scaffold for localized delivery of oxygen. The sustained supply of oxygen was confirmed to enhance wound healing of diabetic mice, along with the EVs to stimulate angiogenesis. On Day 14, the antioxidant polyurethane itself could lead to ~98% wound closure, with the non-diabetic wound closure at 90% and diabetic wound closure at only ~80%. With both the oxygen release and EV delivery functions, the wound closing function of the scaffold could reach ~100% after 14 days for both non-diabetic and diabetic mice. 

In the field of bone tissue regeneration, specific elements can improve the angiogenesis function of the material so as to stimulate the recovery of injured bones. Li^+^ has been proven to promote proliferation and osteogenesis. Liu and colleagues doped LiCl in bioglass ceramics to carry BMSC-EVs^[112]^. The investigation showed that the existence of Li^+^ in the bioglass could significantly improve angiogenesis through the proliferation of HUVECs both *in vitro* and *in vivo* compared to Li^+^-free bioglass by stimulating the activation of Wnt/*β*-catenin, AKT and NF-*κ*B signaling pathways. The addition of proangiogenic miRNA-130a in EVs further activated the PTEN/AKT signaling pathway to enhance the proangiogenic capacity of endothelial cells. The synergic effect brought promise to the material for the vascularization of injured bone tissue.

Despite the above designs, however, the incorporation of extra functions has not been applied extensively in the overall studies of EV delivery systems. Along with EVs, the combinatorial and multimodal benefits towards injuries are to be expected in future experiments.

## CONCLUSION AND PERSPECTIVES FOR FUTURE DESIGN OF EV DELIVERY SYSTEMS

In this review, we surveyed the EV delivery systems from the viewpoint of the material design at the molecular level, focusing on the relationship of material structures, the corresponding physicochemical properties and the performance of EV delivery. Based on the loading and delivery of EVs, the systems were classified into bulk materials, with crosslinking as the crucial structural parameter, and scaffolds, with porosity as the symbolic structure and surface property as the key parameter to interact with EVs. From the analyses of the according delivery behavior *in vitro* and *in vivo*, it can be seen that the delivery can be adjusted in correlation to the changes of material at the molecular level to a certain extent. These all guarantee that the EV delivery systems fill the two “gaps” in the field: one is the successful, localized and sustained release of EVs in the injured tissue; the other is how the material designs can direct the delivery of EVs. From the stand of patients, the use of EV delivery systems can greatly reduce the pain patients receive from otherwise multiple times of systemic, large-dose administration of EVs. This can further lead to cost reduction due to higher efficiency of EV delivery, and better regenerative results due to the various functions different materials can bring about. Therefore, the use of EV delivery systems has shown much promise in promoting EVs as a potential therapeutic approach for tissue regeneration. However, still at its initial stage, the EV delivery systems have missing links within the relationship of material structures, physicochemical properties and the performance of delivery. In order to achieve on-demand control over the delivery behavior through fine-tuning of the materials at the molecular level, we propose and emphasize that EV delivery systems can be further studied in the following directions:

(1) More studies necessary for a better understanding of the impacts of molecular designs on EV delivery

As mentioned in the introduction section, we extracted related information from representative investigations to analyze the relationship between the release profiles *in vitro*, retention of EVs *in vivo* and the resulting tissue regeneration. More systematic studies should be conducted on various types of EV delivery systems for the validation of results, as mentioned above. New reports on the behavior of EV delivery systems should be tracked to build a larger and more complete platform for EV delivery systems;

(2) The interaction between the materials and EVs during the EV storage period

The storage period is one of the three periods of EVs in material delivery systems, but is a missing link compared to the loading and delivery of EVs. Although the results of the delivery systems suggest no obvious change in the function of EVs after a short period of storage, no investigation has been made on how the material affects the encapsulated or carried EVs in the long run. For bulk materials, the distribution of crosslinking junctions/salt crystals and of EVs, as mentioned in “IMPACT OF DIFFERENT DESIGNS ON THE LOADING AND DELIVERY OF EVs”, are critical to the release profile of the EVs and the kinetics of interaction with the recipient cells. Similarly, for scaffold materials, the different surface properties, including mechanical properties, hydrophilicity/hydrophobicity, surface charge, and uninvestigated surface topographies, can also affect the loading and delivery performance of the systems. Meanwhile, previous studies^[140,141]^ have shown that surface properties can affect the attached stem cells to secrete EVs with different cargoes. It can be inferred that as the functional replicates of the parental stem cells, different properties of materials, including both bulk ones and scaffolds, may also affect the behavior of EVs encapsulated or attached. On the basis of the understanding of the influence of materials on the EVs, better selection of materials and processing strategies can be made for future development of the delivery systems;

(3) The kinetics of EV delivery and the internalization of cells by the adjustments of material designs

As was shown by Henriques-Antunes *et al.* on the relationship between the EV release profiles and the healing of diabetic chronic wounds using photo-cleavable crosslinkers in EV delivery hydrogel, the kinetics of EV delivery is crucial to their interactions with cells and wound healing^[136]^. In order to optimize the delivery behavior of the material, careful detection of the relationship between the rates of delivery and the dosage being internalized is necessary. This can be realized, in resemblance to and more controllable than the photo-cleavable crosslinker, by a smart delivery system to finely adjust the interaction between the material and the EVs for release;

(4) Extension to materials previously investigated in tissue engineering

The current exploitation of materials for EV delivery systems accounts for only a small portion of materials in tissue engineering. Many different materials, especially for drug delivery systems, have been developed and extensively investigated. The EV delivery systems can be adapted from these material systems, which will provide a more diverse choice and functions. Furthermore, the scope of material can be extended to fully synthetic materials. Synthetic free radical polymers, for example, have fine structures and can be accurately controlled with living/controlled polymerization techniques. Through the applications such as ring-opening single unit monomer insertion techniques^[142]^ and anionic ring-opening polymerization (polycarbonate series)^[143]^, the non-degradability of free radical polymers has also been solved for the dynamic building of bioactive polymers. The incorporation of these well-defined polymers into EV delivery systems can be of great promise.

## DECLARATIONS

### Copyright

© The Author(s) 2022.
